# Research and Design of Energy-Harvesting System Based on Macro Fiber Composite Cantilever Beam Applied in Low-Frequency and Low-Speed Water Flow

**DOI:** 10.3390/ma17123033

**Published:** 2024-06-20

**Authors:** Rui Huang, Jingjing Zhou, Jie Shen, Jing Tian, Jing Zhou, Wen Chen

**Affiliations:** 1 State Key Laboratory of Advanced Technology for Materials Synthesis and Processing, School of Materials Science and Engineering, Wuhan University of Technology, Wuhan 430070, China; 18672338768hr@sina.com (R.H.); zhoujingjing@whut.edu.cn (J.Z.); shenjie@whut.edu.cn (J.S.); tian.jing@whut.edu.cn (J.T.); 2 Key Laboratory of Functional Materials and Devices for Informatics of Anhui Education Institutes, Fuyang Normal University, Fuyang 236037, China; 3 Sanya Science and Education Innovation Park, Wuhan University of Technology, Sanya 572024, China; 4 Hubei Longzhong Laboratory, Xiangyang 441000, China

**Keywords:** macro fiber composite (MFC), low-frequency, low-speed, energy harvesting

## Abstract

In nature, lakes and water channels offer abundant underwater energy sources. However, effectively harnessing these green and sustainable underwater energy sources is challenging due to their low flow velocities. Here, we propose an underwater energy-harvesting system based on a cylindrical bluff body and a cantilever beam composed of a macro fiber composite (MFC), taking advantage of the MFC’s low-frequency, lightweight, and high piezoelectric properties to achieve energy harvesting in low-frequency and low-speed water flows. When a water flow impacts the cylindrical bluff body, it generates vibration-enhanced and low-frequency vortices behind the bluff body. The optimized diameter of the bluff body and the distance between the bluff body and the MFC were determined using finite element analysis software, specifically COMSOL. According to the simulation results, an energy-harvesting system based on an MFC cantilever beam applied in a low-frequency and low-speed water flow was designed and prepared. When the diameter of the bluff body was 25 mm, and the distance between the bluff body and MFC was 10 mm and the maximum output voltage was 22.73 V; the power density could reach 0.55 mW/cm^2^ after matching the appropriate load. The simulation results and experimental findings of this study provide valuable references for designing and investigating energy-harvesting systems applied in low-frequency and low-speed water flows.

## 1. Introduction

With the development of low-energy electronic products such as integrated circuits, MEMS, and microsensors, there are many drawbacks to energy harvesting and storage methods, including their high cost, large size, and the requirement to replace them frequently [1,2,3,4]. Therefore, there is an urgent need for a sustainable energy supply. In nature, there are many green and sustainable sources of vibrational energy in the environment, making the collection and sustainable utilization of vibrational energy in the environment an increasingly popular research focus [5,6,7,8,9].

Fluid-induced vibration is a common natural phenomenon in which vibrations are stimulated by a force generated by a fluid flow around a bluff body [10,11,12,13]. Flow-induced vibrations encompass various types such as vortex-induced vibration (VIV), galloping, wake-induced vibration (WIV), chatter, and buffeting. In recent years, notable progress has been achieved in the field of energy harvesting through fluid-induced vibration, encompassing the study of wind-induced vibration and water-induced vibration [14,15,16,17]. In a study of wind-induced vibration, Eugeni et al. [18] proposed a flag-flutter-based piezoelectric (PZT) energy harvester to capture energy from wind-induced chatter. Sun et al. [19] proposed a lightbulb-shaped bluff body that increased the wind energy collection rate by 193%. Liu et al. [20] introduced side-by-side double plates in front of the bluff body to amplify the amplitude of the energy harvester, achieving an output voltage of 12 V. Furthermore, fluid-induced vibration in water has also received significant attention from researchers. For example, Javad [21] investigated the impact of the ratio of the bluff body to spacing on energy capture efficiency and found that the maximum power output depends on the frequency of eddy currents and tip displacement. An et al. [22] introduced a novel type of underwater energy harvester which achieved an output voltage of 20 mV under high-Reynolds-number conditions. Gong et al. [23] utilized a curved beam to collect energy from underwater oscillations, resulting in a peak output of 3.28 V. Furthermore, over the past three years, progress in underwater-energy-harvesting research has also been compared and analyzed, as shown in Table 1. Currently, underwater energy-harvesting approaches mainly utilize piezoelectric or magnetostrictive materials, with a greater focus on high-speed water flow research. However, due to limitations in material sensitivity, more research is needed on collecting low-frequency, low-speed, and weak vibration energy.

However, 71% of the Earth’s surface is covered by oceans, and most natural water bodies have low-frequency and low-velocity water flows [29,30,31,32]. Therefore, harnessing low-frequency and low-velocity water flows has been a persistent challenge. Macro fiber composites (MFCs) are sandwich-like structures with high piezoelectricity and low resonant frequencies, enabling them to respond to low-frequency and weak vibration signals. Moreover, MFCs exhibit excellent flexibility, making them suitable for underwater vibration environments. Inspired by the aforementioned research, we propose the application of MFCs in low-frequency and low-velocity water flows. We design an underwater vortex-forming device that utilizes the impact of water flow on a flow-blocking body to induce vortex-induced vibrations for energy harvesting. To maximize the energy collection efficiency of MFCs, this work employs finite element analysis software (COMSOL 6.0) to simulate and analyze the influence of different structural designs on the underwater electrical output performance of an MFC. Based on the simulation results, experimental tests are optimized and conducted, demonstrating excellent energy-harvesting performance. These research findings hold promise for providing new methods to achieve green and sustainable energy-harvesting applications in low-frequency and low-velocity water flow environments.

## 2. Materials and Methods

### 2.1. The System Construction of Energy Harvesting

**Figure 1:**
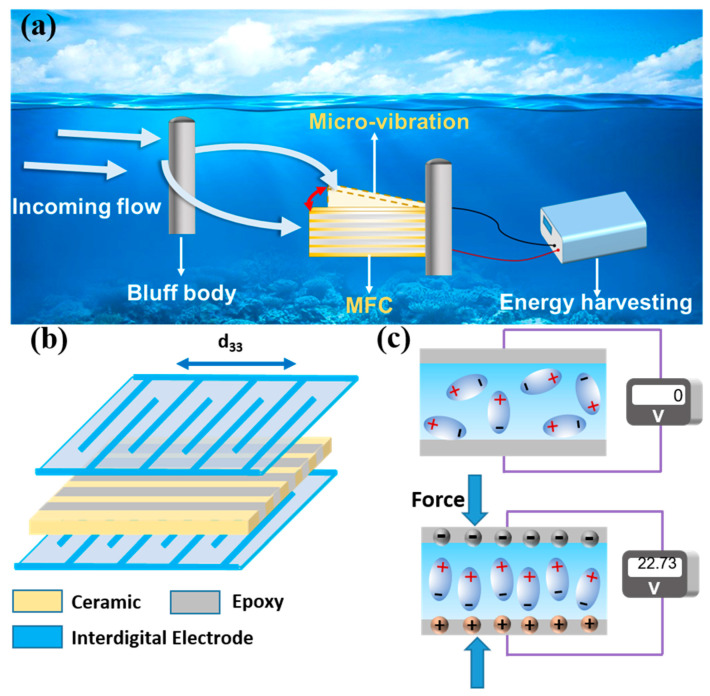
Low-frequency and low-velocity underwater energy harvesting using MFCs: (**a**) system design, (**b**) d_33_ operating mode of MFC, (**c**) and mechanism of energy harvesting. Red arrow represents the vibration direction of the MFC.

This paper presents the design of an underwater energy-harvesting system based on macro fiber composites for energy harvesting in low-frequency and low-velocity water flows, as shown in Figure 1a. The energy-harvesting system consists of an energy storage device, an MFC cantilever beam for energy harvesting, and a flow-blocking body. The MFC cantilever beam is vertically positioned in the low-velocity water flow, with the flow-blocking body placed in front of the MFC. When the water flow impacts the flow-blocking body, vortex-induced forces that can excite vibrations are generated behind the flow-blocking body. Due to an alteration in the water flow caused by the bluff body, two phenomena occur. On one hand, periodic low-frequency vortex-induced vibrations are formed behind the bluff body. On the other hand, an increase in water flow velocity and a subsequent amplification of underwater pressure occur downstream of the bluff body. These vortex-induced forces act on the aluminum substrate cantilever beam of the MFC component, inducing low-frequency micro-vibrations in the MFC. The vibrational energy from the water flow is then converted into electrical energy through the piezoelectric effect of the MFC and outputted to the energy-harvesting system.

The primary sensor device in the energy-harvesting system is the macro fiber composite (MFC), which comprises thin composite layers in a sandwich configuration covering interdigitated electrodes [33]. The intermediate layer of the composite thin layers serves as the functional layer, encompassing piezoelectric ceramic fibers and polymer fibers [34,35]. This work used a PZT-5H piezoelectric ceramic (technical data are shown in Table 2) and DP460 (3M Co. Ltd., St. Paul, MN, USA) to prepare piezoelectric fiber composite sheets. Following a finite element analysis to optimize dimensional parameters in MFC fabrication, the optimal dimensional parameters for MFC were selected, as delineated in Table 3. The MFC can operate in various modes, such as d_33_, d_31_, and d_15_. Among these modes, the d_33_ mode achieves higher energy-harvesting efficiency by utilizing the piezoelectric effect in the length direction of the MFC [36], as depicted in Figure 1b. In this work, the MFC operating in the d_33_ mode is chosen as the transducer device to achieve better energy-harvesting performance. The MFC is affixed to an aluminum substrate to construct a cantilever beam device. When the MFC is not subjected to vortex-induced forces, charges in the piezoelectric ceramic are randomly distributed. However, when the MFC experiences vortex-induced forces generated by the underwater flow, it undergoes reciprocating vibrations, leading to fixed and ordered charge distributions in the electrode arrays and generating an alternating voltage, as shown in Figure 1c. The energy generated from the MFC is rectified and stored for further utilization.

The experimental platform consists of a flow channel, water pump, piezoelectric cantilever beam structure, and energy-harvesting system. The flow channel employs an open channel, with the piezoelectric cantilever beam vertically fixed along the channel’s midline. The free end of the cantilever beam faces the bluff body and the channel inlet, fully submerged in water. After ensuring the waterproof sealing of the cantilever beam wires, they are led out and connected to the energy-harvesting circuit.

A flow channel model with a width of 40 mm, featuring a reservoir at both ends (Figure S1), positions the water pump fixed to the rear reservoir and the pump outlet fixed to the front reservoir, enabling water circulation within the channel. By adjusting the water pump’s intake, the flow velocity within the channel is controlled. Water flows from the front reservoir into the channel inlet and passes through the bluff body to create vortices, which alternately impact the free end of the MFC cantilever beam. The water then exits the channel at the outlet, flows into the rear reservoir, and is pumped back to the front reservoir, forming a circulation system that simulates a low-frequency and low-velocity water flow.

A bluff body is placed 50 mm from the channel inlet, with an MFC cantilever beam energy-harvesting device positioned behind it. The piezoelectric cantilever beam device is waterproofed using a thermoplastic tube encapsulation method. The wires of the MFC cantilever beam are connected to a DMM 7510 digital multimeter to test the output voltage and current. The circuit is tested by adjusting the external resistance using a resistor box. The output of the MFC cantilever beam is rectified and connected to a supercapacitor for energy harvesting, enabling the testing of the output voltage of the piezoelectric cantilever beam.

### 2.2. The Theory of Energy Harvesting

**Figure 2:**
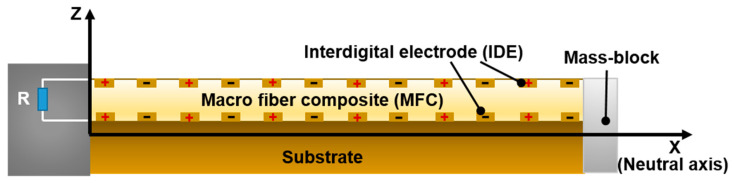
Theoretical analysis and modeling of MFC.

In order to further understand the electrical output performance of the MFC, an analysis of the electromechanical coupling of the MFC is conducted [37,38,39,40]. Additionally, a cantilever beam model for MFC energy harvesting is established, as shown in Figure 2.

According to the Euler–Bernoulli theory, the relationship between the axial strain and the curvature radius of a laminated beam is given by Equation (1).

(1) $$S_{3}\left(x,z,t\right)=-z\frac{\partial^{2}w_{\mathrm{rel}}\left(x,t\right)}{\partial x^{2}}$$

where $w_{\mathrm{rel}}\left(x,t\right)$ represents the transverse displacement of the neutral axis of the beam relative to a fixed reference frame, and $S_{3}\left(x,z,t\right)$ is the strain in the x-direction. The vibration control equation is shown in Equation (2).

(2) $$\frac{\partial^{2}M\left(x,t\right)}{\partial x^{2}}+c_{s}I\frac{\partial^{5}w_{\mathrm{rel}}\left(x,t\right)}{\partial x^{4}\partial t}+c_{a}\frac{\partial w_{\mathrm{rel}}\left(x,t\right)}{\partial t}+m\frac{\partial^{2}w_{\mathrm{rel}}\left(x,t\right)}{\partial t^{2}}=\left[-m+M_{t}\delta \left(x-L\right)\right]\frac{\partial^{2}w_{b}\left(x,t\right)}{\partial t^{2}}-c_{a}\frac{\partial w_{b}\left(x,t\right)}{\partial t}$$

where $M\left(x,t\right)$ represents the bending moment inside the beam, $c_{s}I$ is the structural elastic equivalent damping, $c_{a}$ is the air damping coefficient, m is the mass of the beam, $M_{t}$ is the mass block weight at the end of the cantilever beam used to adjust the resonance frequency, and L is the length of the cantilever beam. According to further derivation, the coupled mechanical equation for the MFC sensor is shown in Equation (3).

(3) $$\begin{array}{l} \mathrm{YI}\frac{\partial^{4}w_{\mathrm{rel}}\left(x,t\right)}{\partial x^{2}}+c_{s}I\frac{\partial^{5}w_{\mathrm{rel}}\left(x,t\right)}{\partial x^{4}\partial t}+c_{a}\frac{\partial w_{\mathrm{rel}}\left(x,t\right)}{\partial t}+m\frac{\partial^{2}w_{\mathrm{rel}}\left(x,t\right)}{\partial t^{2}}+KV\left(t\right)\left[\frac{d\delta \left(x\right)}{dx}-\frac{d\delta \left(x-L\right)}{dx}\right]\\ \;\;\;\;\;=\left[-m+M_{t}\delta \left(x-L\right)\right]\frac{\partial^{2}w_{b}\left(x,t\right)}{\partial t^{2}}-c_{a}\frac{\partial w_{b}\left(x,t\right)}{\partial t} \end{array}$$

where YI is the equivalent stiffness of the MFC cantilever beam, K is the electromechanical coupling coefficient of the MFC, and $V\left(t\right)$ represents the output voltage of the MFC. According to structural mechanics, the control equation for the undamped free vibration of the cantilever beam is shown in Equation (4).

(4) $$\mathrm{YI}\frac{\partial^{4}w_{\mathrm{rel}}\left(x,t\right)}{\partial x^{2}}+m\frac{\partial^{2}w_{\mathrm{rel}}\left(x,t\right)}{\partial t^{2}}=0$$

By using the modal superposition method, the transverse displacement of the piezoelectric cantilever beam can be expressed as Equation (5).

(5) $$w_{\mathrm{rel}}\left(x,t\right)=\sum_{r=1}^{\infty }\phi_{r}\left(x\right)\eta_{r}\left(x\right)$$

where $\phi_{r}\left(x\right)$ represents the r-order mode function, and $\eta_{r}\left(x\right)$ is the r-order mode coordinate. By applying boundary conditions, the natural frequency of the r-order mode can be determined using Equation (6).

(6) $$w_{r}=\lambda_{r}^{2}\sqrt{\mathrm{YI}/L^{4}n}$$

By further derivation, the modal force coefficient $f\left(r\right)$ can be obtained, as shown in Equation (7).

(7) $$f\left(r\right)=-[m\int_{0}^{L}\phi_{r}\left(x\right)d\left(x\right)+Mt\phi_{r}\left(L\right)]$$

Then, the output voltage can be obtained, as shown in Equation (8).

(8) $$V_{0}=\frac{\sum_{r=1}^{\infty }\frac{K}{C_{p}}\frac{jm\omega^{3}f_{r}\omega_{0}}{\omega_{r}^{2}+\omega^{2}+2j\xi \omega_{r}\omega }}{\sum_{r=1}^{\infty }\frac{K}{C_{p}}\frac{j\omega K}{\omega_{r}^{2}-\omega^{2}+2j\xi \omega_{r}\omega }+\frac{1}{RC_{p}}+j\omega }$$

Finally, the expression for the sensing voltage is derived, as shown in Equation (9).

(9) $$V(t)=V_{0}e^{j\omega }$$

## 3. Simulation Results

### 3.1. Effect of Bluff Body Distance on Energy Harvesting

In order to clarify the primary influencing factors and their impact on the underwater energy-harvesting performance of the MFC, a simulation analysis was conducted using the finite element analysis software COMSOL to examine the underwater pressure distribution on the MFC. The simulation depicted the bluff body as a white, circular shape and the MFC as a white, elongated rectangle.

**Figure 3:**
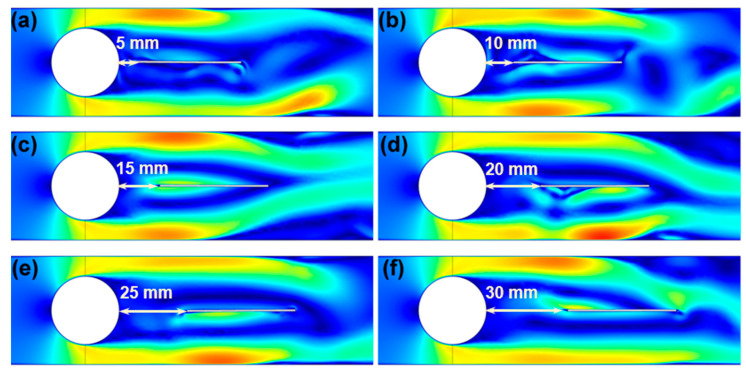
Influence of different distances on MFC energy harvesting: (**a**) 5 mm, (**b**) 10 mm, (**c**) 15 mm, (**d**) 20 mm, (**e**) 25 mm, and (**f**) 30 mm.

The pressure distribution of water on the MFC after passing through the bluff body was analyzed. Figure 3 illustrates the impact of varying distances between the bluff body and the MFC on the pressure distribution in the water surrounding the MFC. The left side of the MFC cantilever beam corresponds to the free end, whereas the right side represents the fixed end. When a vortex is generated on the right side of the bluff body, the resulting vortex-induced vibration pressure is fully applied to the fixed end of the cantilever beam, leading to a higher voltage output and, consequently, more effective energy harvesting. Upon comparing the results, it was observed that when the distance between the bluff body and the MFC was 10 mm, the maximum loading of the vortex-induced vibration occurred at the fixed end of the MFC, leading to a significant electrical output effect.

### 3.2. Effect of Bluff Body Diameter on Energy Harvesting

**Figure 4:**
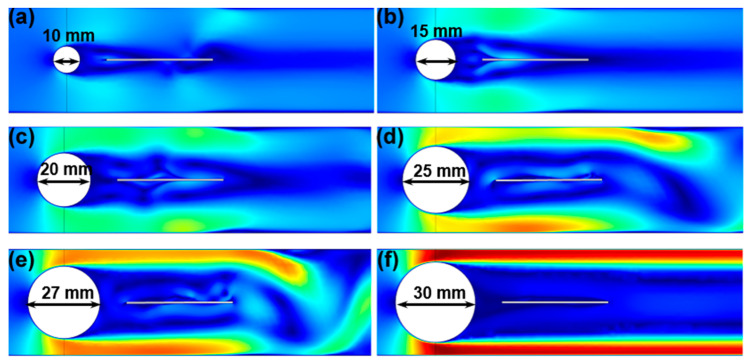
Influence of different diameters on MFC energy harvesting: (**a**) 10 mm, (**b**) 15 mm, (**c**) 20 mm, (**d**) 25 mm, (**e**) 27 mm, and (**f**) 30 mm.

A flow channel (600 mm × 40 mm × 70 mm) was constructed for the purpose of facilitating testing by simulating a low-speed water flow environment. Figure 4 illustrates the influence of the diameter of the bluff body on the pressure distribution in the water surrounding the MFC. It presents pressure distribution on the MFC for bluff body diameters ranging from 10 mm to 30 mm. The results revealed that as the diameter of the bluff body increased, the pressure between the bluff body and the channel on the MFC also increased. This phenomenon can be attributed to the formation of a high-pressure “wind tunnel effect” in the narrow region between the channel and the bluff body. However, when the bluff body diameter exceeds a certain threshold, as shown in Figure 4f, the water flow becomes parallel to the channel direction, resulting in the absence of vortex formation on the MFC and the inability to induce vortex-induced vibration.

Through a comparison of pressure distributions for various bluff body diameters, it is evident that the highest pressure exerted by the vortex on the cantilever beam occurs when the MFC diameter is 25 mm. Additionally, the vortex pressure is relatively low for bluff body diameters of 10 mm, 15 mm, and 20 mm. No vortex formation is observed on the MFC for bluff body diameters of 27 mm and 30 mm. Consequently, a bluff body diameter of 25 mm is deemed the most suitable choice for energy-harvesting purposes.

## 4. Experiment Results and Discussion

**Figure 5:**
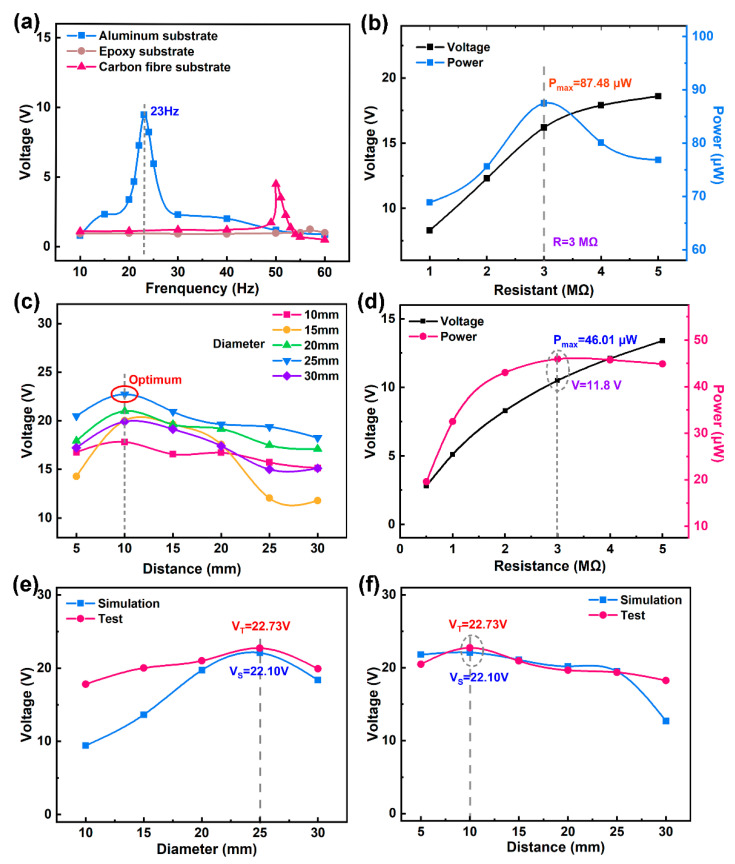
The electrical output performance of the MFC: (**a**) the output voltage of the MFC on different substrates, (**b**) the energy-harvesting performance of the MFC cantilever beam with an aluminum substrate, (**c**) the influence of MFC energy harvesting, (**d**) the influence of the parallel resistor on MFC energy harvesting, (**e**) a comparison of simulated and tested output voltages for different bluff body diameters, and (**f**) a comparison of simulated and tested output voltages for different distances between the bluff body and the MFC.

The underwater vibration environment was simulated using a vibration table (SCU-200, Su Shi Testing Group Co., Ltd, Suzhou, China), the voltage performance of the MFC cantilever beam was tested using a digital multimeter (7510, Tektronix, Beaverton, OR, USA), and load performance was tested using a variable resistance box, as illustrated in Figure 5a,b. Underwater testing was simulated using an exciter, as shown in Figure 5a, and the MFC showed exceptional electrical output performance on an aluminum substrate, an epoxy substrate, and a carbon-fiber board when subjected to an applied excitation force of 4 m/s^2^ acceleration. Among these substrates, the MFC cantilever beam on the aluminum substrate exhibited the lowest resonant frequency (23 Hz) and the highest output voltage of 10.1 V. It can be seen that when aluminum-based MFCs are used for energy harvesting, they exhibit minimal energy loss and maximize the outputted electrical signal. Figure 5b shows the energy-harvesting performance of the MFC aluminum substrate cantilever beam device with different external resistors. As the resistance increased, the output voltage of the MFC gradually increased, while the output electrical power initially increased and then decreased. When the resistor was set to 3 MΩ, the MFC achieved a maximum output power of 87.48 μW. It is evident that MFCs exhibit high sensitivity to environmental stimuli, making them well-suited for low-frequency and low-velocity water flow environments. It can be seen that the MFC fabricated with an aluminum substrate exhibited good energy-harvesting performance in terms of its low frequency, high power, and high sensitivity, making it well-suited for low-frequency and low-velocity underwater energy-harvesting applications.

The MFC was utilized in a self-assembled underwater energy-harvesting system, and a water flow rate of about 0.5 m/s was pumped to evaluate its performance when collecting energy underwater, as shown in Figure 5c–f. From Figure 5c, it can be observed that the output open-circuit voltage of the MFC exhibits an initial increase followed by a decrease as the distance between the bluff body and the MFC increases, and when the distance is equal to 10 mm, the energy acquisition systems with different bluff body diameters all reach the maximum voltage. When comparing the output voltages for different diameters of the bluff body, it is evident that the maximum output voltage of 22.73 V is attained when the bluff body diameter is 25 mm. Figure 5d shows the energy-harvesting performance of the underwater MFC system when varying the parallel connection of different output resistors. As the resistance value increases, the output voltage exhibits an upward trend, whereas the output power initially rises and subsequently declines. When the parallel resistance is equal to the internal resistance of the MFC (3 MΩ), the output voltage of the MFC is 11.8 V with a maximum power of 46.01 μW, corresponding to a maximum power density of 0.55 mW/cm^2^.

Upon comparing the simulation results for underwater energy harvesting using the MFC with the experimental findings, it is apparent that the trend in the energy-harvesting voltage for various bluff-body diameters remains consistent between the simulation and test results. As shown in Figure 5e, the output voltage demonstrates an increasing–decreasing pattern with an increase in diameter. Notably, when the bluff body diameter is 25 mm, both the simulation and experimental results indicate the presence of a maximum open-circuit voltage, measuring 22.10 V and 22.73 V, respectively. Furthermore, Figure 5f illustrates the impact of different distances between the bluff body and the MFC on the MFC’s output voltage, with the simulation trend closely aligning with the experimental trend. Specifically, at a distance of 10 mm, both the simulation and test result outcomes indicate the attainment of the maximum MFC output open-circuit voltage, measuring 22.10 V and 22.73 V, respectively. It is evident that in the designed low-frequency underwater energy-harvesting system with the optimized blunt body diameter and position, the output electrical signal of the aluminum-based cantilever-beam-structured MFC energy harvester is the highest, enabling stable voltage outputs of above 22 V underwater. Additionally, the experimental value slightly surpasses the simulated value due to the variable water velocity.

## 5. Conclusions

To harness the green energy sources present in lakes and water channels in nature and achieve sustainable energy harvesting in low-frequency and low-speed water flows, we propose an underwater energy-harvesting system based on an MFC. This system takes advantage of the MFC’s low-frequency, lightweight, and high piezoelectric properties. Finite element analysis software was utilized to determine the optimal values for the diameter of the bluff body and the distance between the bluff body and the MFC. Subsequently, a testing platform was constructed to evaluate the energy-harvesting system. The obtained results are as follows:

1. The finite element design results indicate that the diameter of the bluff body and the position have a significant impact on the capture efficiency, with vortices only being generated with appropriate dimensions. In a vortex environment, low-frequency vortex-induced forces are formed, and the amplified vortex-induced forces increase the water pressure to achieve high capture efficiency. A diameter of 25 mm generates the strongest vortex pressure and is the most suitable for energy harvesting. And at a distance of 10 mm, the vortex pressure is maximized and can effectively act on the MFC cantilever beam.
2. According to the theoretically calculated influence law, a low-frequency low-speed underwater energy-harvesting system was designed and prepared. The maximum voltage is 22.73 V and the maximum power density is 0.55 mW/cm^2^.

Further exploration is required to determine how to achieve broadband and high-efficiency energy harvesting using MFCs underwater. This will be the focus of our future research.

## Figures and Tables

**Table 1:** Comparison of underwater energy-harvesting methods.

Materials	Fluid Form	Flow Velocity (m/s)	Frequency (Hz)	Output Voltage (V)	Output Power (μW)	Ref.
TENG	sea wave	--	--	--	20	[24]
PVDF cantilever	water flow	--	--	--	0.38	[25]
piezoelectric–electromagnetic material	sea wave and wind	18.36	0.4	20.34	--	[26]
PZT–magnetic coupling	tide	--	--	13.6	--	[27]
MFC cantilever	water flow	--	--	0.132	--	[28]
MFC cantilever	water flow	0.5	20	22.73	46.01	This work

**Table 2:** Technical data for PZT piezoelectric ceramic.

Piezoelectric Constant d (pC/N)	Density (kg/m^3^)	Dielectric Constant
$d_{31}$ $d_{33}$ $d_{15}$	ρ	$\varepsilon_{11}^{E}$ $\varepsilon_{33}^{E}$
330 372 380	7450	919 827

**Table 3:** Technical specifications of MFC.

Component	Parameter	Symbol	Units	Value
Piezoceramic fiber array	fiber width	W_f_	mm	0.35
	fiber spacing	S_f_	mm	0.35
	fiber thickness	T_f_	mm	0.30
Interdigitated electrode (IDE)	IDE finger width	I_w_	mm	0.10
	IDE finger spacing	I_s_	mm	0.50
	IDE finger thickness	I_t_	mm	0.04

## Data Availability

The raw data supporting the conclusions of this article will be made available by the authors on request.

## Supplementary Materials

The following supporting information can be downloaded at: https://www.mdpi.com/article/10.3390/ma17123033/s1, Figure S1: MFC energy harvesting system (a) System construction, (b) System detail; Figure S2: Schematic diagram of operation mode for MFC.
