# Supplementary material for: Research and Design of Energy-Harvesting System Based on Macro Fiber Composite Cantilever Beam Applied in Low-Frequency and Low-Speed Water Flow
Source: Materials (Basel). 2024 Jun 20;17(12):3033. doi: 10.3390/ma17123033 (PMC11205516; doi:10.3390/ma17123033)
Supplement: Supplementary file 1 [file materials-17-03033-s001.zip › materials-2999500-supplementary.pdf]

1. System construction

The experimental platform consists of a flow channel, water pump, piezoelectric cantilever beam structure, and energy harvesting system. The flow channel employs an open channel, with the piezoelectric cantilever beam vertically fixed along the channel's midline. The free end of the cantilever beam faces the bluff body and the channel inlet, fully submerged in water. After ensuring the waterproof sealing of the cantilever beam wires, they are led out and connected to the energy harvesting circuit.

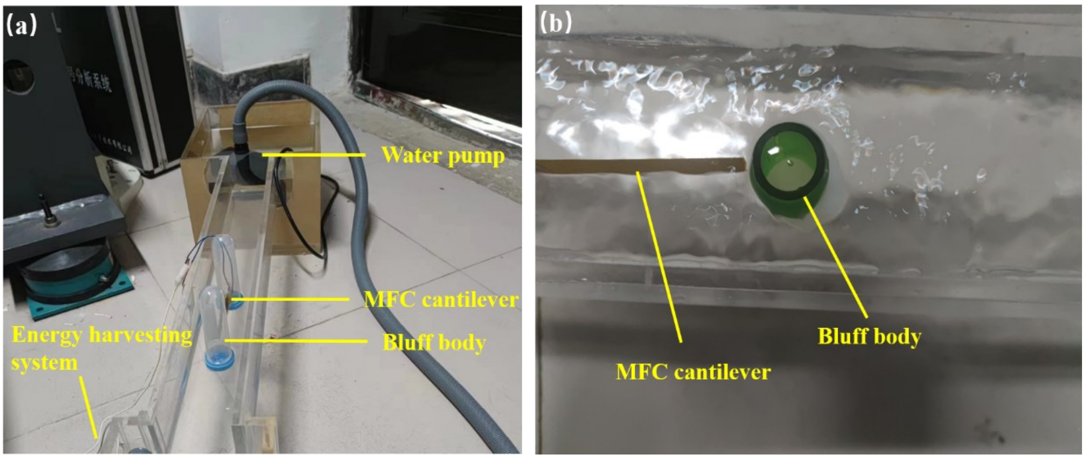

Figure S1. MFC energy harvesting system (a) System construction, (b) System detail

A flow channel model with a width of 40mm, featuring a reservoir at both ends (Figure S1), positions the water pump fixed to the rear reservoir and the pump outlet fixed to the front reservoir, enabling water circulation within the channel. By adjusting the water pump's intake, the flow velocity within the channel is controlled. Water flows from the front reservoir into the channel inlet and passes through the bluff body to create vortices, which alternately impact the free end of the MFC cantilever beam. The water then exits the channel at

the outlet, flows into the rear reservoir, and is pumped back to the front reservoir, forming a circulation system that simulates low-frequency and low-velocity water flow.

2. MFC operating mode

According to the direction of polarization and the direction of the applied electric field, the working modes of MFC are d_{33}, d_{31}, d_{15}, etc. The d_{33} is one of the piezoelectric (strain) constant matrix parameters.d_{33} type MFC uses the d_{33} effect to make MFC work in d_{33} operation mode, d_{31} type MFC uses the d_{31} effect to make MFC work in d_{31} operation mode and d_{15} type MFC uses the d_{15} effect to make MFC work in d_{15} operation mode. Figure S2 is the picture of the MFC electromechanical coupling operation mode. The driving electric field direction and polarization direction remain parallel in the two modes. Among them, the d_{33} operation mode is mainly designed for the axial driving performance of the piezoelectric ceramic fiber. The driving electric field direction and polarization direction are periodically and non-uniformly distributed along the axial direction of the fiber. The work in the d_{33} operation mode can effectively play the axial piezoelectric performance of piezoelectric ceramic fiber, which is the focus of this paper.

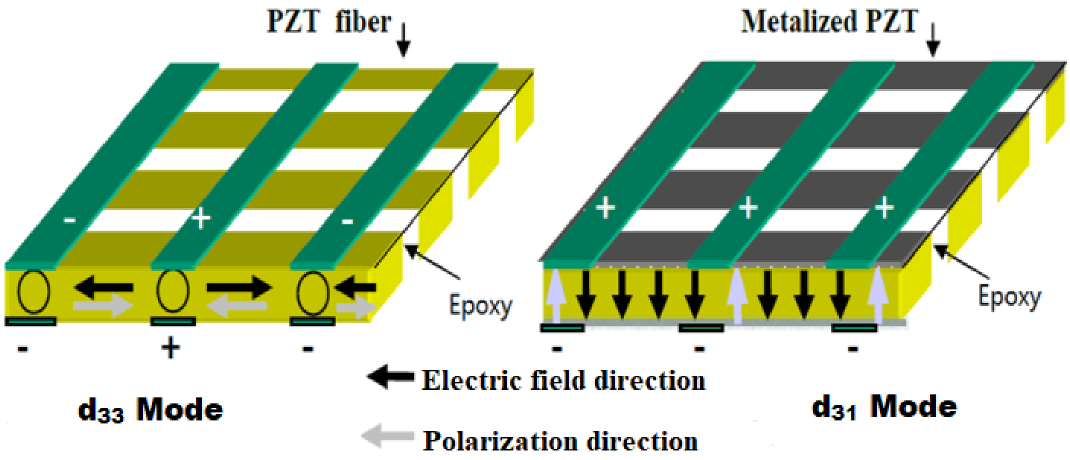

Figure S2. Schematic diagram of operation mode for MFC
